# Some Forms of Tertiary Syphilis

**Published:** 1898-07-09

**Authors:** J. A. Bloxam

**Affiliations:** Senior Surgeon to the Hospital


### Medical Progress and Hospital Clinics.

[*The Editor will be glad to receive offers of co-operation and contributions from members of the profession. All letters should be addressed to* The Editor, at the Office, 28 & 29, Southampton Street, Strand, London, W.C.]

### SOME FORMS OF TERTIARY SYPHILIS.

A Clinical Lecture given at Charing Cross Hospital by J. A. Bloxam, F.R.C.S., Senior Surgeon to the Hospital.

A case was shown of a man, aged 45, whose history was the following: That his left testicle had been enlarged during the last two years. Five years previously he had a chancre, followed by sore throat, for which he was treated for only three weeks.

It was pointed out that there were gummatous patches on the arms, face, and legs, which were ulcerating, and that the patient had a deep sepulchral voice with thickening of the parts about the larynx, and that he was deaf. The left testicle was three times the size of the right one, and was covered in front by a large slough; the discharge—which was not very profuse—was from round the edge of the slough, and it had a most offensive smell; the scrotum around the edge of the slough was of a bright red colour. The testicle felt hard, smooth, and very heavy; the epididymis could not be distinguished from the body of the organ. When the testicle was squeezed it was noticed that testicular sensation was lost, this loss of sensation being very characteristic of syphilitic disease. There was no thickening of the tissues of the scrotum, no enlarged veins, and no thickening of the vas deferens.

On examining the right testicle it was shown that there was also slight hardness and enlargement. This condition was pointed out to be due to a chronic inflammation resulting in a deposit of small round cells between the tubules of the testicle; this deposit of cells contracts, presses on the tubules, and, unless removed by treatment, destroys them. But if the case is treated before contraction has occurred these cells may be removed, and the case may recover without destruction of the tubules; therein differing from tuberculous disease, in which the tubes are generally destroyed, the result being impotency, while in syphilis the recovery may be perfect. The testes may be the seat of syphilic disease in early infant life, the result of hereditary disease, and then they present much the same appearance.

Hydrocele is often described as being common in conjunction with a syphilitic testicle. This was against the lecturer's experience, he having noticed that the cavity of the tunica vaginalis was often obliterated by adhesions. The tunica albuginea is sometimes irregular and nodular, but this is not usual; but it frequently becomes greatly thickened.

The two forms in which syphilis appears to attack the testes are (1) in the diffuse form, or (2) in patches forming masses of gummata, but they are both the result of precisely the same process. In the gummatous on section they present the appearance of yellow wash-leather masses. In the diffuse the whole body of the testes presents that appearance.

What are the points that would enable us to distinguish between this condition and other common forms of testicular enlargement? In enlargement following chronic orchitis there is generally some history of an acute stage, but in syphilis this is never the case; it comes on without any reason apparent to the patient. In acute, painful gonorrhœal orchitis there is always some flattened shape of the testicle, whereas in syphilis it is distinctly globular. In other forms we have the history of gout, rheumatism, or urethral discharge. Again, in chronic orchitis the disease begins in the epididymis, but in syphilis the disease begins in the testicle itself, which enlarges, and eventually, so to speak, swallows up the epididymis. Could the condition be due to tubercle? No; for this usually starts in the epididymis, and is associated with the thickened cord, which is not the case here, the cord being normal. Could this case be one of sarcoma or adenoma? It will be seen not to be such on noting these points. The rate of growth (which in syphilis is slow and in sarcoma and adenoma rapid—weeks or months); in this testis the enlargement has been coming on for two years. In sarcoma the epididymis and spermatic cord are involved, the veins in the scrotum and spermatic cord are enlarged and tortuous, and if the disease has attacked the skin the glands in groin appear involved. In this case the cord is normal, glands are not affected in the groin, nor does any thickening of the cord or vas appear.

Regarding the treatment, it was remarked that in all forms of late manifestations of syphilis when suppuration is present mercury should not be given, but that potassium iodide could be given in doses of from 10 up to 60 grains, and this even three times a day with the best result. This patient was taking 20 grains of potassium iodide three times a day. As there is always a certain amount of debility present in these cases 1 fl. dr. of tincture of cinchona and 5 grains of ammonium carbonate were added.

Regarding the local treatment of cases like the above, the shortest and best way is to remove the testicle; but most patients object to this treatment. When admitted there was a large slough on the scrotum, and no line of demarkation between the slough and the scrotum existed; however, this was already taking place; unguentum hydragari was applied locally, but no mercury was given by the mouth.

The history of this case showed what frequently happens after an imperfect course of treatment. He appeared to have had no treatment of any kind for more than three weeks, and then concluded that he was cured; being a sailor, and in vigorous health, no syphilic symptoms appeared for a time, now they were present in the worst forms, namely, ulceration of patches of skin gummata, with the same disease attacking important organs such as the larynx and testis. In the lecturer's experience treatment in any case of syphilis should extend to two years, even when no active symptoms are present.

It was pointed out that the patches on the forearm were simple ulceration of gummata of skin, and that the scar on the forearm had a brownish stain with a serpiginous edge; that the scars on the face were very white; and that this extreme whiteness of the scars was very characteristic of syphilis. In the treatment of syphilis, the importance of the patient being temperate, with early hours and a simple diet, was insisted on, the lecturer concluding with the saying that the combination of syphilis and drink was utterly incurable.

